# Baltimore Medical and Surgical Journal

**Published:** 1850-08

**Authors:** 


Dr. Hull, of Baltimore is about to issue a new monthly paper, to be entitled the "Baltimore Medical and Surgical Journal."

